# Intraoperative Cardiac Events During Pediatric Cardiac Magnetic Resonance Imaging Under Anesthesia: A Retrospective Cohort Study

**DOI:** 10.1002/pan.70249

**Published:** 2026-06-19

**Authors:** Theodora Wingert, Justine Liang, Kelly Feldman, Amelie Delaporte, Christine Nguyen‐Buckley, Tristan Grogan, Alexandre Joosten, John Paul Finn, Ihab Ayad

**Affiliations:** ^1^ Department of Anesthesiology & Perioperative Medicine, David Geffen School of Medicine at UCLA University of California Los Angeles Los Angeles California USA; ^2^ Division of Critical Care Medicine, Department of Pediatrics University of California Los Angeles Los Angeles California USA; ^3^ Department of Anesthesiology University of California Los Angeles Los Angeles California USA; ^4^ Department of Medicine Statistics Core, David Geffen School of Medicine University of California Los Angeles Los Angeles California USA; ^5^ Department of Radiology, David Geffen School of Medicine University of California Los Angeles Los Angeles California USA

**Keywords:** hypotension, intraoperative cardiac events, mortality, pediatric, perioperative complications

## Abstract

**Background:**

Children with congenital heart disease (CHD) frequently undergo cardiac magnetic resonance imaging (MRI) under anesthesia. Although these procedures lack surgical stimulus, patients remain physiologically vulnerable, and the incidence of perioperative cardiovascular instability in this setting is poorly defined. We aimed to determine the incidence and predictors of intraoperative cardiac events during pediatric cardiac MRI using a recently proposed composite framework of intraoperative cardiovascular instability.

**Methods:**

This single‐center retrospective cohort study analyzed patients ≤ 18 years undergoing cardiac MRI under anesthesia from 2013 to 2025. The primary outcome was intraoperative cardiac events, defined as ≥ 5 cumulative minutes with mean arterial pressure > 2 standard deviations below age‐ and sex‐adjusted reference values, vasopressor administration, or severe adverse cardiac events. Multivariable logistic regression identified independent predictors. Thirty‐day mortality was evaluated descriptively.

**Results:**

Among 330 cases, intraoperative cardiac events occurred in 29.7% (98/330). Hypotension was most common (24.2%), followed by vasopressor administration (9.7%); no severe adverse cardiac events were observed. Thirty‐day mortality was 1.8% (6/330). Independent predictors included increasing age (OR 1.13 per year, 95% CI: 1.04–1.21), and higher‐acuity preoperative location, including neonatal intensive care unit (ICU) (OR 4.42, 95% CI: 1.86–10.52) and pediatric ICU (OR 4.84, 95% CI: 1.91–12.26). Sensitivity analyses demonstrated consistent findings across model specifications.

**Conclusions:**

Intraoperative cardiac events, primarily hypotension of unknown significance, were common during pediatric cardiac MRI under anesthesia; no severe adverse cardiac events occurred. Our findings highlight the physiologic complexity of this population and support the need for thoughtful perioperative risk stratification and management.

## Clinical Implications

- What is already known
  ○ Pediatric patients with congenital heart disease undergoing cardiac MRI under anesthesia are a vulnerable group; however, the frequency and nature of intraoperative cardiovascular instability remain poorly characterized.
- What new information this study adds
  ○ In this study, nearly one‐third of children experienced an intraoperative cardiac event, predominantly hypotension, yet no severe adverse cardiac events occurred. These findings suggest that cardiac MRI under anesthesia appears generally safe in an experienced tertiary pediatric medical center while revealing frequent hemodynamic perturbations whose physiologic and clinical significance remain to be determined.

## 1 Introduction

Congenital heart disease (CHD) affects approximately 1% of live births, making CHD one of the most common congenital conditions [1, 2, 3]. Advances in pediatric cardiology, cardiac surgery, anesthesia, and intensive care have improved survival, resulting in a growing population with complex physiology who require repeated diagnostic and interventional procedures throughout childhood [2, 3, 4]. As survival improves, optimizing perioperative safety in this vulnerable population has become increasingly important.

Cardiac magnetic resonance imaging (MRI) has emerged as a powerful and sometimes indispensable diagnostic modality in pediatric cardiology. It can provide high‐resolution anatomic detail in three dimensions or higher, ventricular volumetrics, unique functional information, tissue characterization, and quantitative flow assessment that may not be possible from other non‐invasive modalities. However, high quality cardiac MRI in young children typically requires general anesthesia and mechanical ventilation for optimal imaging. Delivering anesthesia in the MRI environment presents unique challenges, including limited patient access, physical separation of the anesthesia provider from the patient, restricted availability of equipment, reliance on MR‐conditional monitoring and infusion systems, and remote location away from anesthesia support [5]. These factors may increase the risk of delayed recognition and management of physiologic changes, as well as delays in access to additional anesthesia or clinical support due to the remote location.

Children with CHD undergoing noncardiac surgery have higher rates of perioperative complications compared to children without CHD [6]. In the Pediatric Health Information System cohort, postoperative mortality in children with CHD undergoing noncardiac surgery was about 10 times that of non‐CHD patients [7]. In a recent study examining the incidence of hypotension undergoing noncardiac surgery, children with CHD were not observed to have significantly higher rates of intraoperative hypotension [8]. Most studies in children with CHD undergoing noncardiac procedures have focused on mortality as the primary outcome, with relatively few examining the incidence, risk factors, and intraoperative management of perioperative events.

Recently, Nasr and colleagues reported the incidence of *intraoperative cardiac events* in a large multicenter registry of children with CHD undergoing noncardiac procedures, defining *events* as a composite of hypotension, vasoactive medication use, and severe adverse cardiac events. This framework conceptualizes perioperative cardiovascular instability as a spectrum rather than isolated catastrophic outcomes and standardizes risk characterization in pediatric cardiac populations [9]. Despite the growing use of cardiac MRI [10], the incidence and predictors of intraoperative cardiac events during MRI performed under anesthesia are poorly described. Understanding patterns of cardiovascular instability in this setting is essential for perioperative planning, resource allocation, and discussions with families.

The primary aim of this study was to determine the incidence of intraoperative cardiac events in children undergoing cardiac MRI with anesthesia at a tertiary medical center using the previously described intraoperative cardiac event composite framework. The secondary aim was to identify factors independently associated with intraoperative cardiac events.

## 2 Methods

After IRB approval (IRB#24‐000495), we retrospectively evaluated patients ≤ 18 years who underwent cardiac MRI with anesthesia at Ronald Reagan UCLA Medical Center from 2013 to 2025. Cardiac MRI combined procedures (such as catheterization lab) and cases without anesthesia were excluded. All cases were performed at the institution's tertiary pediatric hospital campus with onsite pediatric intensive care, pediatric cardiology, pediatric cardiac surgery, and dedicated pediatric anesthesiology and pediatric cardiac anesthesiology support. At our institution, anesthesiology staffing for pediatric patients with congenital heart disease is guided by internal case coverage guidelines, with cases staffed by pediatric or pediatric cardiac anesthesiology teams based on patient and procedural characteristics. The manuscript was prepared according to STROBE guidelines.

### 2.1 Primary Outcomes

The primary outcome was the incidence of intraoperative cardiac events, which was defined as a composite of intraoperative hemodynamic instability and severe adverse cardiac events, in keeping with prior literature [9]. Intraoperative cardiac events were defined as the presence of: (1) ≥ 5 cumulative minutes with mean arterial pressure (MAP) > 2 standard deviations (SD) below age‐ and sex‐adjusted reference under anesthesia [8], or (2) bolus or infusion vasopressors, or (3) severe adverse cardiac events, defined as intraoperative cardiac arrest, pulmonary hypertensive crisis, air embolism, or arrhythmia [9, 11]. The presence of severe adverse cardiac events was determined from the electronic anesthesia record, including intraoperative free‐text notes, and from a mandatory quality reporting database.

For this study, we defined intraoperative hypotension as a cumulative intraoperative duration with MAP > 2 SD below age‐ and sex‐adjusted reference for 5 or more minutes [11, 12]. Intraoperative blood pressures were recorded electronically automatically from anesthesia start to anesthesia stop every 3–5 min via intermittent noninvasive blood pressure monitoring or every minute via an invasive arterial line (if present); if both modalities were present, arterial blood pressures were utilized. If a value was not recorded at a given minute, the last known MAP was carried forward until a new value was recorded (“last observation carried forward” method), following conventions used in previous large‐scale perioperative studies [13]. Also in keeping with current methodologies, if no blood pressure measurement was recorded for more than 5 consecutive minutes, a maximum of 5 min was counted toward the cumulative exposure duration [13, 14]. Artifacts were excluded using predefined thresholds, namely if MAP < 10 or > 200 mmHg and systolic blood pressure < diastolic blood pressure.

Other studies have utilized absolute reduction or percentages from baseline blood pressure, however this approach may be influenced by falsely elevated preinduction measurements related to patient distress or limited cooperation and by challenges in establishing a reliable baseline value [9, 15]. Additionally, preinduction baseline blood pressure measurements were frequently unavailable in our cohort, as these could be missing if no blood pressure was recorded prior to the induction event time, or if the induction event time was not charted. To improve data completeness, a composite preoperative blood pressure variable was constructed. When preinduction blood pressure measurements were unavailable, the median of the three most recent preoperative blood pressure values obtained within 24 h before the procedure was used.

Use of age‐ and sex‐adjusted blood pressure references from healthy non‐CHD children, as applied in this study, has been reported in prior investigations [12, 16]. This reference has been utilized in studies including children with CHD, but not to our knowledge in cohorts primarily consisting of CHD patients [8, 12]. The applicability of this reference in CHD population is not well established and no CHD‐specific normative standards currently exist. We pre‐specified a threshold of 2 SD below age‐ and sex‐adjusted reference values to identify clinically meaningful hypotension while minimizing misclassification due to expected physiologic variability [8]. We used medication data from the EHR to obtain intraoperative vasopressor bolus and infusion information. Vasopressors were defined as epinephrine, norepinephrine, phenylephrine, dopamine, ephedrine, or vasopressin [9].

Mortality was defined as any death occurring within 30‐days after cardiac MRI captured by the UCLA EHR, identified by discharge tables and death notes and tables within the UCLA Perioperative Data Warehouse [17].

### 2.2 Perioperative Characteristics

Patient demographics and perioperative variables were extracted from the EHR using our departmental Perioperative Data Warehouse [17]. Preoperative patient characteristics included age, sex, height, weight, American Society of Anesthesiologists (ASA) status, medications, and laboratory values. Intraoperative variables included type of anesthetic, duration of anesthesia, intraoperative medications administered, and minute‐level blood pressure and pulse oximetry data. Type of anesthesiology provider specified whether the anesthesiologist was pediatric cardiac subspecialty trained versus pediatric anesthesiology trained only; anesthesia trainee and nurse anesthetist involvement was also recorded. All “quick notes” from the anesthesia records, which are free text notes describing intraoperative events, were reviewed as a sensitive way to capture adverse events. Intraoperative oxygen desaturation was defined as two or more cumulative minutes with oxygen saturation less than 70% in patients with cyanotic physiology, and as SpO_2_ < 90% for at least 2 cumulative minutes in non‐cyanotic patients [18]. Cyanotic physiology was defined as congenital heart disease associated with systemic desaturation, including right‐to‐left shunt, single‐ventricle, or ductal‐dependent physiology [19]. Preoperative location was modeled as a categorical variable rather than dichotomized, as we sought to preserve clinically meaningful distinctions between care settings and to assess whether risk differed across specific locations.

Cardiac disease was categorized using the American College of Surgeons (ACS) National Surgical Quality Improvement Program (NSQIP) classification, which classifies CHD into minor, major, or severe [20]. Minor CHD is defined as a cardiac condition with or without medication and maintenance (e.g., atrial septal defect or small‐to‐moderate ventricular septal defect without symptoms) or a repaired CHD with normal cardiovascular function and no medication. Major CHD is defined as a repaired CHD with residual hemodynamic abnormality with or without medications. Severe CHD is defined as uncorrected cyanotic heart disease, any documented pulmonary hypertension, ventricular dysfunction requiring medications, or listed for heart transplant. Several additional variables to characterize cardiac disease severity were also captured, including preoperative prostaglandin E (PGE) (indicating ductal‐dependent lesions), pulmonary hypertension history and medications, history of heart transplant, preoperative supplemental oxygen, preoperative vasopressor or inotropic support (including milrinone), and presence of cyanotic physiology (discussed above) [21].

### 2.3 Nomogram Figure

To facilitate clinical interpretation, the final multivariable logistic regression model was translated into a nomogram figure. The nomogram converts each predictor in the model into a standardized point scale, with the total point sum corresponding to an estimated probability of an intraoperative cardiac event. This approach allows patient‐level risk estimation by summing points across predictors and mapping the total to a predicted event probability. Continuous variables, such as age, are represented directly on the scale, avoiding categorization, and all predictors are displayed on a common metric to enable visual comparison of their relative contributions.

### 2.4 Statistical Methods

Patient characteristics and study variables were summarized overall and then by group (e.g., presence of intraoperative cardiac events or 30‐day mortality) using means with SD for continuous variables and frequencies with percentages for categorical variables. Group comparisons were performed using *t*‐tests or ANOVA for continuous variables and chi‐square or Fisher's exact tests for categorical variables. Multivariable logistic regression identified independent predictors of intraoperative cardiac events. Covariates were selected based on clinical relevance. Given the small sample size and reduction in sample size with missing data as more variables were added, only a small number of variables were included within the multivariable models. With the final cohort of 330 cases, with an approximately 2:1 split between patients without versus with the primary composite outcome, this provided approximately 80% power to detect a difference in proportions as small as 16% (e.g., 42% vs. 58% using a chi‐square test, *α* = 0.05) for categorical predictors and approximately 80% power to detect a standardized mean difference of 0.35 for continuous predictors, using a two‐sided *t*‐test, alpha of 0.05. The primary outcome occurred in 98 patients, supporting a parsimonious multivariable logistic regression model with a limited number of clinically selected covariates. The final model included approximately 12 events per parameter. Analyses of 30‐day mortality were descriptive only because only 6 deaths occurred. Results are presented as odds ratios (OR) with 95% confidence intervals (CI). All analyses were conducted using R version 4.4.3 (R Foundation for Statistical Computing, Vienna, Austria). A two‐sided *p*‐value < 0.05 was considered statistically significant.

### 2.5 Sensitivity Analyses

To assess the robustness of variable selection, we fit a sensitivity model that included additional covariates with low rates of missing data (prematurity, endotracheal tube in situ, and anesthesia duration), while excluding candidate variables with high levels of missing data (e.g., pre‐induction MAP). We performed sensitivity analyses modeling hypotension and vasopressor use as separate outcomes to evaluate their relative contributions to the composite endpoint. In addition, we conducted sensitivity analyses including year of surgery as a covariate to assess for potential temporal trends.

## 3 Results

Over a 12‐year period, 1431 pediatric cardiac MRIs were performed. After excluding cardiac MRI cases combined with procedures (*N* = 220) and those performed without anesthesia (*N* = 881), 330 cardiac MRIs with anesthesia in patients age ≤ 18 years were analyzed. Preoperative characteristics of the cohort undergoing cardiac MRI with anesthesia are shown in Table 1 and intraoperative characteristics are shown in Table 2.

**TABLE 1:** Patient characteristics by intraoperative cardiac event.

	Overall (*N* = 330)	Intraoperative cardiac event		*p*
		No (*N* = 232)	Yes (*N* = 98)	
Age	3.8 (4.2)	3.8 (3.8)	3.8 (5.0)	0.987
Sex (M)	171 (51.8%)	127 (54.7%)	44 (44.9%)	0.102
Weight (kg)	16.6 (11.0)	16.4 (10.9)	18.4 (13.5)	0.737
ASA score				
1	2 (0.6%)	1 (0.4%)	1 (1.0%)	0.169
2	46 (14.1%)	36 (15.7%)	10 (10.4%)	
3	195 (59.8%)	141 (61.3%)	54 (56.2%)	
4	83 (25.5%)	52 (22.6%)	31 (32.3%)	
CHD NSQIP category				
No CHD	11 (3.3%)	10 (4.3%)	1 (1.0%)	0.047
Mild	29 (8.8%)	23 (9.9%)	6 (6.1%)	
Major	98 (29.7%)	75 (32.3%)	23 (23.5%)	
Severe	193 (58.2%)	124 (53.4%)	68 (69.4%)	
Cyanotic physiology	137 (41.5%)	83 (35.8%)	54 (55.1%)	0.001
Prematurity (y/n)	39 (11.8%)	26 (11.2%)	13 (13.3%)	0.597
Preop suppl O_2_	39 (11.8%)	25 (10.8%)	14 (14.3%)	0.367
Preop PGE (y/n)	117 (35.5%)	72 (31.0%)	45 (45.9%)	0.01
Preop hemodialysis (y/n)	25 (7.6%)	14 (6.0%)	11 (11.2%)	0.104
History of heart transplant (y/n)	9 (2.7%)	5 (2.2%)	4 (4.1%)	0.459
History of pulmonary HTN (y/n)	31 (9.4%)	18 (7.8%)	13 (13.3%)	0.117
Preop endothelin antagonist (y/n)	21 (6.4%)	11 (4.7%)	10 (10.2%)	0.063
Prior cath lab procedure (y/n)	181 (54.8%)	120 (51.7%)	61 (62.2%)	0.079
Prior cardiac surgery procedure (y/n)	196 (59.4%)	132 (56.9%)	64 (65.3%)	0.155
Preoperative location				
Outpatient	215 (65.2%)	169 (72.8%)	46 (46.9%)	< 0.001
Floor	22 (6.7%)	13 (5.6%)	9 (9.2%)	
NICU	51 (15.5%)	27 (11.6%)	24 (24.5%)	
PCTU	12 (3.6%)	7 (3.0%)	5 (5.1%)	
PICU	30 (9.1%)	16 (6.9%)	14 (14.3%)	
Postoperative				
30‐Day mortality	6 (1.8%)	4 (1.7%)	2 (2.0%)	1
Postop ICU	114 (34.5%)	68 (29.3%)	46 (46.9%)	0.002
Postop cath lab procedure (y/n)	126 (38.2%)	79 (34.1%)	47 (48.0%)	0.017
Postop cardiac surgical procedure (y/n)	156 (47.3%)	99 (42.7%)	57 (58.2%)	0.01

*Note:* Data are presented as mean (SD) or median (Q1, Q3) for continuous variables, and number (percent) for categorical variables.

Abbreviations: ASA, American Society of Anesthesiologists; CHD NSQIP, Congenital Heart Disease National Surgical Quality Improvement Program; CI, confidence interval; HTN, hypertension; loc, location; MAC, monitored anesthesia care; NICU, neonatal intensive care unit; PCTU, pediatric cardiothoracic unit; PGE, prostaglandin E; PICU, pediatric intensive care unit; SD, standard deviation.

**TABLE 2:** Intraoperative characteristics.

	Overall (*N* = 330)	Intraoperative cardiac event		*p*
		No (*N* = 232)	Yes (*N* = 98)	
Anesthesia type				
General	305 (98.3%)	210 (98.1%)	95 (99.0%)	1
MAC	5 (1.6%)	4 (1.9%)	1 (1.0%)	
Anesthesia duration (min)	144.9 (42.5)	145.9 (42.1)	142.8 (43.4)	0.554
Procedure duration (min)	59.9 (28.3)	60.0 (25.9)	59.6 (32.7)	0.915
Arterial line	25 (7.6%)	15 (6.5%)	10 (10.2%)	0.241
Midazolam (y/n)	30 (9.1%)	16 (6.9%)	14 (14.3%)	0.033
Intraop dexmedetomidine (y/n)	20 (6.1%)	17 (7.3%)	3 (3.1%)	0.138
Morphine equivalent (mg/kg)	0.1 (0.2)	0.1 (0.2)	0.1 (0.2)	0.220
Any vasopressor or inotrope (y/n)	32 (9.7%)	0	32 (32.7%)	< 0.001
Any vasopressor or inotrope infusion (y/n)	9 (2.7%)	0	9 (9.2%)	< 0.001
Oxygen desaturation over 2 min (y/n)	104 (31.5%)	66 (28.4%)	38 (38.8%)	0.065
ETT in situ (y/n)	34 (13.5%)	20 (11.8%)	14 (16.9%)	0.272
Remained intubated end of case (y/n)	58 (23.0%)	137 (81.1%)	57 (68.7%)	0.028
Hemodynamics				
Preoperative MAP (median, mmHg)	67.5 (16.6)	68.0 (17.0)	66.3 (15.7)	0.422
Duration MAP < 1 SD (min)	51.5 (37.0)	39.2 (30.6)	70.5 (38.1)	< 0.001
Median (Q1, Q3)	51.0 (18.0, 79.8)	33.5 (11.0, 62.2)	73.0 (47.2, 91.5)	
Duration MAP < 2 SD (min)	21.1 (24.3)	2.5 (1.0)	28.4 (25.1)	< 0.001
Median (Q1, Q3)	11.0 (3.0, 33.0)	3.0 (2.0, 3.0)	17.5 (10.0, 42.0)	
Anesthesia practice characteristics				
Attending anesthesia staffing				
Pediatric cardiac anesthesiologist	44 (13.3%)	29 (12.5%)	15 (15.3%)	0.493
Pediatric anesthesiologist	286 (86.7%)	203 (87.5%)	83 (84.7%)	
Anesthesia resident/fellow involved (y/n)	208 (63.0%)	137 (59.1%)	71 (72.4%)	0.021
CRNA involved (y/n)	17 (5.2%)	15 (6.5%)	2 (2.0%)	0.097
Any handoff (y/n)	23 (7.0%)	13 (5.6%)	10 (10.2%)	0.134

*Note:* Data are presented as mean (SD) or median (Q1, Q3) for continuous variables, and number (percent) for categorical variables. Oxygen desaturation was defined as SpO_2_ < 70% in patients with cyanotic physiology, and as SpO_2_ < 90% for at least 2 cumulative minutes in non‐cyanotic patients.

Abbreviations: CI, confidence interval; CRNA, certified registered nurse anesthetist; ETT, endotracheal tube; MAC, monitored anesthesia care; MAP, mean arterial pressure; PGE, prostaglandin E; SBP, systolic blood pressure; SD, standard deviation.

The overall incidence of intraoperative cardiac events in our study was 29.7% (98/330) (Table 3). Of these 98 cases, intraoperative cardiac events in the form of hypotension alone were observed in 24% and hypotension plus vasoactive medication was observed in 9.7% (31/330). No severe adverse cardiac events occurred. Of the 31 patients who were documented as receiving vasopressors, most (22/31) received boluses only, including 12 who received a single bolus. Nine patients received a continuous infusion, most commonly epinephrine. Among cases with ≥ 5 cumulative minutes of BP > 2 SD below reference values without vasopressor use, the median duration was 17 min. Among patients with hypotension who did receive vasopressors, the median duration below the MAP threshold was 25.5 min; the difference between patients with hypotension who received vasopressors versus those who did not was not statistically significant, *p* = 0.299. Thirty‐day mortality occurred in 1.8% (6/330). The mean age was 3.8 years (median 2 years). Twenty‐five cases (7.6%) had arterial catheters for invasive blood pressure monitoring. Almost all cases were general anesthetics (98.3%) with 1.7% monitored anesthesia care (MAC). All general anesthetics utilized volatile‐based maintenance anesthesia. No cases with general anesthesia with an airway utilized propofol total intravenous anesthesia as the primary anesthetic technique. All general anesthetics with an airway were performed with endotracheal tube (ETT) airways (no laryngeal mask airways were utilized), 34 (10.3%) had an ETT in situ, and 58 (23%) remained intubated at the end of the case. Most cases (86.7%) were cared for by pediatric anesthesiologists and the rest (13.3%) were cared for by pediatric cardiac anesthesiologists.

**TABLE 3:** Intraoperative cardiac event incidence.

Event component	*N* = 330	Percent
Hypotension‐only	67/330	20.3
Hypotension plus vasopressor	31/330	9.4
Severe adverse cardiac event	0/330	0
Any intraoperative cardiac event	98/330	29.7

*Note:* Any intraoperative cardiac event was defined as a composite of three components: (1) intraoperative hemodynamic instability involving hypotension‐only, (2) intraoperative hemodynamic instability involving hypotension plus vasopressor medication use, and (3) severe adverse cardiac events. Hypotension was defined as ≥ 5 cumulative minutes with mean arterial pressure (MAP) > 2 standard deviations (SD) below age‐ and sex‐adjusted reference under anesthesia. Vasopressor medication use was defined as intraoperative bolus or infusion vasopressor. Severe adverse cardiac events were defined as intraoperative cardiac arrest, pulmonary hypertensive crisis, air embolism, or arrhythmia.

Breakdown by CHD classification showed the majority of cases to have Major (29.7%) or Severe CHD (58.2%). Additionally, 137 (41.5%) had cyanotic physiology, 117 (35.5%) were on a PGE infusion, 39 (11.8%) were on preoperative supplemental oxygen, overall 35% were inpatients (of these 15.5% from the neonatal intensive care unit [NICU], 12.7% from the pediatric intensive care unit [PICU] or pediatric cardiothoracic unit [PCTU]), 6.7% from the floor settings, and the majority had prior cardiac catheterization (54.8%) and cardiac surgery (59.4%).

Univariate analyses demonstrated CHD classification, PGE (45.9 vs. 31%; *p* = 0.01), preoperative location, cyanotic physiology (55.1 vs. 28.4%, *p* = 0.001), midazolam (14.3 vs. 6.9%; *p* = 0.033), and anesthesia trainee involvement were significantly associated with cardiac events compared to those without. Univariate analyses also demonstrated patients with intraoperative cardiac events were more likely to remain intubated at the end of the case (31.8 vs. 18.9%; 0.028), and require the intensive care unit (ICU) after the procedure (49.6 vs. 29.3%; *p* = 0.002). Among the 102 patients who experienced an intraoperative cardiac event, 3 (2.9%) had 30‐day mortality, compared with 3 of 228 patients (1.3%) without an intraoperative cardiac event. Of the patients with mortality, all had cyanotic physiology, 4/6 were inpatient neonates, the two outpatients were under 2 years of age. There was no significant difference in 30‐day mortality among those who experienced intraoperative cardiac events versus those who did not (*p* = 0.378), indicating no observed association between intraoperative cardiac events and 30‐day mortality.

Analyses of characteristics associated with mortality were limited to univariable analyses, given the low absolute number of events. Associations of perioperative characteristics and 30‐day mortality are shown in Table S1.

Multivariable adjusted analyses showed increasing age (OR 1.13 per year, 95% CI: 1.04–1.21) and preoperative location were independently associated with higher odds of intraoperative cardiac events in multivariable analyses (Table 4). Preoperative location was significantly associated with intraoperative cardiac events, with increased risk for patients admitted from all higher‐acuity settings, including the hospital floor (OR 3.97, 95% CI: 1.41–11.19), neonatal ICU (OR 4.42, 95% CI: 1.86–10.52), pediatric cardiac ICU (OR 4.47, 95% CI: 1.15–17.4), and pediatric ICU (OR 4.84, 95% CI: 1.91–12.26). Notably, CHD classification, PGE, prematurity, history of pulmonary hypertension, preoperative supplemental oxygen, and 30‐day mortality were not associated with intraoperative cardiac events. However, multivariable statistical analyses were limited by sample size.

**TABLE 4:** Multivariable adjusted logistic regression of intraoperative cardiac events.

Variable	Logistic regression		
	Odds ratio (OR)	95% CI (lower–upper)	*p*
Age (year)	1.13	1.04–1.21	0.002
ASA score (unit)	0.79	0.48–1.28	0.329
CHD NSQIP classification	1.32	0.84–2.09	0.225
Preop PGE	1.48	0.73–3.03	0.277
Preop loc: Floor vs. outpatient	3.97	1.41–11.19	0.009
Preop loc: NICU vs. outpatient	4.42	1.86–10.52	0.001
Preop loc: PCTU vs. outpatient	4.47	1.15–17.40	0.031
Preop loc: PICU vs. outpatient	4.84	1.91–12.26	0.001

Abbreviations: CHD NSQIP, Congenital Heart Disease National Surgical Quality Improvement Program; loc, location; NICU, neonatal intensive care unit; PCTU, pediatric cardiothoracic unit; PGE, prostaglandin E; PICU, pediatric intensive care unit; Preop, preoperative.

**FIGURE 1:**
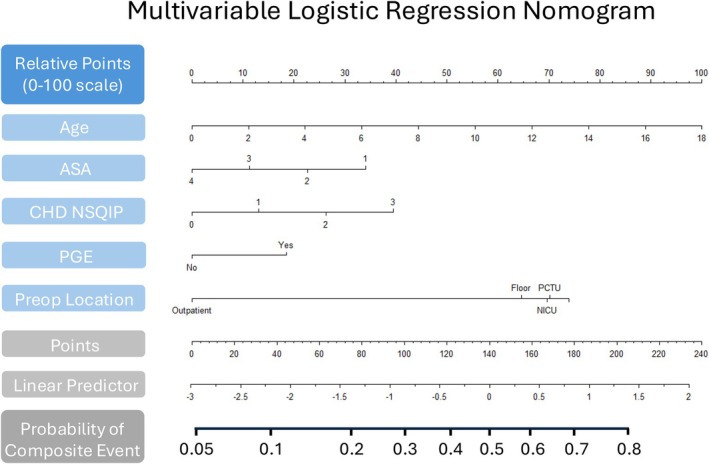
Multivariable logistic regression nomogram. This nomogram is derived from the final multivariable logistic regression model and translates each predictor into a standardized point scale. Points assigned for each variable are summed to generate a total score, which corresponds to a predicted probability of the composite event. The linear predictor represents the log‐odds value from the regression model, and probability of the composite event indicates the corresponding predicted probability of experiencing the composite intraoperative cardiac event. ASA, American Society of Anesthesiologists Physical Status; CHD NSQIP, Congenital Heart Disease National Surgical Quality Improvement Program; PGE, intraoperative prostaglandin E infusion; Preop, preoperative.

Figure 1 displays the nomogram derived from the multivariable model. Each predictor contributes a specified number of points based on its value, and the sum of these points corresponds to an estimated event probability. For example, a hypothetical patient aged 2 years with ASA physical status 3, Severe CHD, no PGE exposure, and an outpatient preoperative location would accumulate approximately 60 total points, corresponding to an estimated event risk of approximately 17%. The nomogram visually demonstrates the relative influence of individual predictors and provides an intuitive framework for translating model outputs into patient‐specific risk estimates.

### 3.1 Sensitivity Analyses

Sensitivity analyses included generation of models including only cases with vasopressor use (with hypotension‐only cases removed). These analyses demonstrated comparable effect estimates for age and location with consistent directionality relative to the primary composite outcome, with the notable difference that CHD classification was significantly associated with intraoperative vasopressor use (OR 3.31; 95% CI: 1.12–9.78) (Table S2). Although odds ratios were similar in magnitude, confidence intervals widened and *p*‐values were attenuated when components were modeled individually (i.e., hypotension alone vs. hypotension plus vasopressor), reflecting smaller event counts and reduced statistical power rather than disproportionate influence of a single component. Sensitivity analyses were also conducted that included year of service, which showed that adjustment for calendar year did not materially alter the results.

Finally, sensitivity analyses evaluating alternative variable selection demonstrated minimal impact from missingness, with a similar sample size (*N* = 319 vs. *N* = 326 in the primary model). The primary predictors, age and preoperative location, demonstrated nearly identical effect sizes and levels of statistical significance compared with the core model. Additional candidate variables, including year of surgery, were not independently associated with the outcome and did not materially improve model discrimination, supporting the preference for a more parsimonious model based on reliably captured preoperative variables.

## 4 Discussion

In this single‐center cohort of 330 children undergoing anesthesia for cardiac MRI, intraoperative cardiac events in the form of hypotension‐alone were observed in 24% of cases and hypotension plus vasoactive medication was observed in 9.7%. No severe adverse cardiac events occurred. Increasing age and higher‐acuity preoperative location were independently associated with intraoperative cardiac events. The clinical significance and interpretability of these hypotensive events are uncertain, as such definitions have not been validated in children with CHD. The observed 30‐day mortality rate of 1.8% underscores the high‐risk profile of this population due to their underlying cardiac disease. Importantly, our findings demonstrate that significant fluctuations in blood pressure frequently occur even during non‐surgical diagnostic procedures.

Unlike surgical procedures, cardiac MRI is performed without surgical stimulation or tissue injury. The high incidence of intraoperative cardiac events observed in this study likely reflects intrinsic cardiovascular vulnerability rather than procedural stress. Anesthetic and narcotic agents may eliminate pre‐procedural stress and reduce sympathetic activity and vasomotor tone, leading to decreases in blood pressure, heart rate, and metabolic demand, which may or may not be associated with decreased perfusion. In children with heart disease in particular, distinguishing between a compensated low‐stress physiologic state and one of impaired cardiac output can be challenging in settings like this. Indeed, because diagnostic procedures are often perceived as lower risk, such anesthetic complexities and monitoring requirements may be underestimated. Nonetheless, it is important that our findings should not be interpreted as evidence that cardiac MRI under anesthesia is unsafe. Severe adverse cardiac events were not observed, and all intraoperative cardiac events were managed by the pediatric anesthesiologist.

Nasr et al. [9] introduced a composite definition of intraoperative cardiac events encompassing hypotension, vasoactive support, and major adverse intraoperative outcomes in children with CHD undergoing noncardiac procedures. In that study, intraoperative cardiac events occurred in approximately 5% of cases, substantially lower than the 30% incidence in our cohort. This difference may in part reflect cohort severity, as only 36% of patients in the Nasr et al. cohort had Major or Severe CHD compared with 88% in our study. As an additional reference point looking specifically at hypotension—a recent single‐center study of children with and without CHD undergoing noncardiac surgery reported hypotension to occur in 7.8% of cases; with hypotension defined as systolic blood pressure below 2 SD from reference values for ≥ 5 min [8].

Several factors likely explain these differences. First, cardiac MRI populations represent a selectively higher‐risk group, with 88.2% of patients in the study classified as Major or Severe CHD. Second, our study leveraged minute‐level blood pressure data extracted directly from the electronic anesthesia record, allowing for increased granularity that may not be captured in multicenter registries relying on aggregate or manually reported data. Third, the MRI environment introduces unique physiologic and operational constraints which may influence both detection and management of hemodynamic instability. Our findings suggest that even during non‐surgical diagnostic procedures, children with CHD frequently experience measurable hemodynamic perturbations requiring active anesthetic management [22, 23].

Indeed, children with CHD may also have lower baseline blood pressure due to underlying physiology or medications. Although there are few studies of hypotension in children with CHD vs. those without, one large single center study observed that hypotension did not occur more frequently in children with CHD versus those without CHD [8]. Defining hypotension in children with CHD is particularly challenging. In children, and particularly those with CHD, the blood pressure threshold required for adequate end‐organ perfusion is not known. To address this limitation in our study, we pre‐specified hypotension at a threshold of 2 SD below age‐ and sex‐adjusted reference values, but the validity of this assumption is not established.

There were several additional notable findings. Increasing age was associated with a higher incidence of intraoperative cardiac events, which may reflect cumulative cardiovascular physiologic burden in patients with longstanding CHD, with progressive divergence from non‐CHD physiology over time. As children with CHD age and progress through staged or palliative repairs, often accumulating additional medications, it is possible that their baseline hemodynamics may increasingly diverge from healthy ASA I–II reference populations [11, 24]. Determining optimal blood pressure for children with CHD represents an important area for future investigation.

Patients undergoing cardiac MRI typically require this imaging modality to provide detailed high‐resolution anatomic detail, precise ventricular volumetrics, tissue characterization (e.g., edema, fibrosis, and scar), quantitative flow and shunt calculations, and assessment of pulmonary venous and systemic blood flow—information that cannot be obtained from echocardiogram or cardiac catheterization [25, 26]. As such, children with CHD undergoing cardiac MRI tend to have particularly complex pathology and few of the patients in this cohort had no CHD or minor CHD [21, 25, 26].

Interestingly, despite studies demonstrating improvements in outcomes over time, although the study period spanned 12 years, we did not observe meaningful temporal variation in outcomes [4]. Another interesting, clinically relevant finding was that all inpatient preoperative locations demonstrated similar effect sizes on the primary outcome. Children admitted from inpatient wards had risk comparable to those from intensive care settings, challenging assumptions that elevated risk is confined to high‐acuity environments.

Several additional limitations should be considered. First, the retrospective single‐center design limits generalizability and introduces potential residual confounding despite multivariable adjustment. Second, the composite outcome was primarily driven by hypotension, while vasopressor administration may partly reflect provider practice patterns rather than uniform physiologic thresholds. Clinicians may also have intervened using non‐vasoactive strategies, such as fluid boluses or reductions in delivered volatile anesthetic concentration; however, these interventions are difficult to reliably capture retrospectively. Third, the small number of mortality events limited statistical power for outcome analyses beyond descriptive comparisons.

Finally, this study was not able to assess the clinical significance of hypotension or the relationship between systemic blood pressure and cardiac output. Blood flow and organ perfusion may increase, decrease, or stay the same as blood pressure fluctuates. Reductions in blood pressure during anesthesia may occur due to the elimination of anxiety and stress relative to the awake state, vasodilation, or depression of myocardial contractility or heart rate. Our study utilized a reference population that did not include children with CHD, which may limit the generalizability of this definition of hypotension to this population. Additional studies are needed to determine reference ranges for children with CHD, as well as studies as to whether threshold‐based approaches are appropriate in diverse pediatric populations. Moreover, measurement artifacts inherent to MR‐conditional monitoring systems may influence blood pressure recordings, although automated artifact filtering and sensitivity analyses yielded consistent findings. Despite these limitations, this study represents one of the largest contemporary cohorts examining anesthesia‐related blood pressure fluctuations and vasoactive support during pediatric cardiac MRI and can serve to fuel debate and research about their physiological significance [27, 28, 29, 30, 31, 32].

Future research includes several emerging technologies in cardiac MRI, such as free‐breathing acquisition techniques and accelerated imaging sequences, which could shorten scan duration and potentially reduce physiologic stress and would be of significant benefit to safety in this high‐risk population [33, 34, 35]. Future work integrating four‐dimensional flow measurements synchronized with blood pressure time stamps may actually enable correlation between systemic blood pressure changes and cardiac output, pulmonary flow, or other perfusion surrogates, which would help clarify the clinical significance, or lack thereof, of intraoperative hypotension in children with CHD.

## 5 Conclusion

During pediatric cardiac MRI under anesthesia, intraoperative cardiac events, primarily in the form of hypotension, were common, whereas serious adverse intraoperative events were not observed. These findings suggest that measurable hemodynamic perturbations frequently occur in this physiologically vulnerable population but are typically transient. The physiologic significance of intraoperative blood pressure changes in children with CHD during cardiac MRI remains uncertain and warrants future investigation incorporating flow‐ and perfusion‐based measures. Cardiac MRI under anesthesia appears generally safe in an experienced tertiary center, while revealing frequent hemodynamic perturbations whose physiologic and clinical significance remain to be determined.

## Funding

During the preparation of this work Dr. Wingert has been supported by a Mentored Research Training Grant (MRTG) from the Foundation for Anesthesia Education and Research (FAER) and the Society of Academic Associations of Anesthesiology & Perioperative Medicine (SAAAPM) and a National Institutes of Health K08 from the National Institute of Biomedical Imaging and Bioengineering (1K08EB038393).

## Supporting information


**Table S1:** 30‐Day mortality univariable analyses.
**Table S2:** Multivariable adjusted logistic regression of hypotension with intraoperative vasopressor administration.

## Data Availability

The data that support the findings of this study are available on request from the corresponding author. The data are not publicly available due to privacy or ethical restrictions.
