# Supplementary material for: Intraoperative Cardiac Events During Pediatric Cardiac Magnetic Resonance Imaging Under Anesthesia: A Retrospective Cohort Study
Source: Paediatr Anaesth. 2026 Jun 19;36(9):1143–52. doi: 10.1002/pan.70249 (PMC13460832; doi:10.1002/pan.70249)
Supplement: Supplementary file 1 [file PAN-36-1143-s001.docx]

Supplementary Table S1. 30-day Mortality Univariable Analyses

|  |  | 30-day Mortality | |  |
| --- | --- | --- | --- | --- |
|  | Overall | No | Yes | P-value |
|  | (N=330) | (N=324) | (N=6) |  |
| Age | 3.8 (4.2) | 3.9 (4.2) | 0.4 (0.7) | 0.041 |
| Sex (M) | 171 (51.8%) | 169 (52.2%) | 2 (33.3%) | 0.434 |
| Weight (kg) | 16.4 (14.8) | 16.6 (14.8) | 4.8 (3.6) | 0.054 |
| ASA Score |  |  |  | 0.5 |
| 1 | 2 (0.6%) | 2 (0.6%) | 0 |  |
| 2 | 46 (14.1%) | 46 (14.4%) | 0 |  |
| 3 | 195 (59.8%) | 192 (60.0%) | 3 (50.0%) |  |
| 4 | 83 (25.5%) | 80 (25.0%) | 3 (50.0%) |  |
| CHD NSQIP Category |  |  |  | 0.356 |
| No CHD | 11 (3.3%) | 11 (3.4%) | 0 |  |
| Mild | 29 (8.8%) | 29 (9.0%) | 0 |  |
| Major | 98 (29.7%) | 98 (30.2%) | 0 |  |
| Severe | 192 (58.2%) | 186 (57.4%) | 6 (100.0%) |  |
| Prematurity (y/n) | 39 (11.8%) | 37 (11.4%) | 2 (33.3%) | 0.150 |
| Preop Suppl O2 | 39 (11.8%) | 37 (11.4%) | 2 (33.3%) | 0.150 |
| Preop Milrinone Infusion | 21 (6.4%) | 19 (5.9%) | 2 (33.3%) | 0.050 |
| Preop Hemodialysis (y/n) | 25 (7.6%) | 25 (7.7%) | 0 | 1.000 |
| History of Heart Transplant (y/n) | 9 (2.7%) | 9 (2.8%) | 0 | 1.000 |
| History of Pulmonary HTN | 31 (9.4%) | 31 (9.6%) | 0 | 1 |
| Preop endothelin antagonist (y/n) | 21 (6.4%) | 21 (6.5%) | 0 | 1 |
| Prior Cath Lab Procedure (y/n) | 181 (54.8%) | 177 (54.6%) | 4 (66.7%) | 0.694 |
| Prior Cardiac Surgery Procedure (y/n) | 196 (59.4%) | 192 (59.3%) | 4 (66.7%) | 1.000 |
| Preoperative Location |  |  |  | <0.001 |
| Outpatient | 215 (65.2%) | 169 (72.8%) | 46 (46.9%) |  |
| Floor | 22 (6.7%) | 13 (5.6%) | 9 (9.2%) |  |
| NICU | 51 (15.5%) | 27 (11.6%) | 24 (24.5%) |  |
| PCTU | 12 (3.6%) | 7 (3.0%) | 5 (5.1%) |  |
| PICU | 30 (9.1%) | 16 (6.9%) | 14 (14.3%) |  |
| Anesthesia Practice |  |  |  |  |
| Pediatric Cardiac Anesthesiologist | 44 (13.3%) | 43 (13.3%) | 1 (16.7%) | 0.579 |
| Anesthesia Resident/Fellow | 208 (63.0%) | 204 (63.0%) | 4 (66.7%) | 1 |
| CRNA | 17 (5.2%) | 17 (5.2%) | 0 | 1 |
| Any Handoff | 23 (7.0%) | 23 (7.1%) | 0 | 1 |
| Intraoperative Characteristics |  |  |  |  |
| Anesthesia Type |  |  |  |  |
| General | 305 (98.4%) | 299 (98.4%) | 6 (100.0%) | 1 |
| MAC | 5 (1.6%) | 5 (1.6%) | 0 |  |
| Anesthesia Duration (min) | 144.9 (42.5) | 144.8 (42.1) | 151.2 (62.5) | 0.718 |
| Procedure Duration (min) | 59.9 (28.3) | 60.1 (28.4) | 42.7 (9.0) | 0.289 |
| Arterial Line | 25 (7.6%) | 22 (6.8%) | 3 (50.0%) | 0.007 |
| PGE (y/n) | 117 (35.5%) | 112 (34.6%) | 5 (83.3%) | 0.023 |
| Midazolam (y/n) | 30 (9.1%) | 30 (9.3%) | 0 | 1.000 |
| Dexmedetomidine (y/n) | 20 (6.1%) | 20 (6.2%) | 0 | 1.000 |
| Morphine Equivalent (mg/kg) | 0.1 (0.2) | 0.1 (0.2) | 0.0 (0.1) | 0.785 |
| Any Vasopressor or Inotrope (y/n) | 32 (9.7%) | 0 | 32 (32.7%) | <0.001 |
| Any Vasopressor or Inotrope infusion (y/n) | 9 (2.7%) | 0 | 9 (9.2%) | <0.001 |
| Oxygen Desaturation over 2min (y/n) | 104 (31.5%) | 100 (30.9%) | 4 (66.7%) | 0.081 |
| ETT in situ (y/n) | 34 (13.5%) | 20 (11.8%) | 14 (16.9%) | 0.272 |
| Remained Intubated end of case (y/n) | 58 (23.0%) | 137 (81.1%) | 57 (68.7%) | 0.028 |
| Hemodynamics |  |  |  |  |
| Preinduction MAP (median, mmHg) | 67.5 (16.6) | 67.5 (16.6) | 68.1 (15.9) | 0.934 |
| Duration MAP <1SD | 51.5 (37.0) | 39.2 (30.6) | 70.5 (38.1) | <0.001 |
| Median (Q1,Q3) | 51.0 (18.0, 79.8) | 33.5 (11.0, 62.2) | 73.0 (47.2, 91.5) |  |
| Duration MAP <2SD | 21.1 (24.3) | 2.5 (1.0) | 28.4 (25.1) | <0.001 |
| Median (Q1,Q3) | 11.0 (3.0, 33.0) | 3.0 (2.0, 3.0) | 17.5 (10.0, 42.0) |  |
| Postoperative |  |  |  |  |
| 30-day Mortality | 6 (1.8%) | 4 (1.7%) | 2 (2.0%) | 1 |
| Postop ICU | 114 (34.5%) | 68 (29.3%) | 46 (46.9%) | 0.002 |
| Postop Cath Lab Procedure (y/n) | 126 (38.2%) | 79 (34.1%) | 47 (48.0%) | 0.017 |
| Postop Cardiac Surgical Procedure (y/n) | 156 (47.3%) | 99 (42.7%) | 57 (58.2%) | 0.01 |
| Data are presented as mean (SD) or median (Q1, Q3) for continuous variables, and number (percent) for categorical variables. Abbreviations: American Society of Anesthesiologists (ASA), hypertension (HTN), congenital heart disease listed in EHR Medical History (CHD Med Hx), confidence interval (CI), standard deviation (SD), location (loc), neonatal intensive care unit (NICU), Pediatric Cardiothoracic Unit (PCTU), Pediatric intensive care unit (PICU), certified registered nurse anesthetist (CRNA), monitored anesthesia care (MAC), prostaglandin E (PGE), oxygen saturation (SpO2), endotracheal tube (ETT), mean arterial pressure (MAP), systolic blood pressure (SBP). | | | | |

Supplementary Table S2. Multivariable Adjusted Logistic Regression of Hypotension with Intraoperative Vasopressor Administration

|  | Logistic regression (Vasopressor) | | |
| --- | --- | --- | --- |
| Variable | Odds Ratio (OR) | 95% CI (Lower–Upper) | p-value |
| Age (year) | 1.18 | 1.05–1.32 | 0.004 |
| ASA Score | 1.55 | 0.69–3.51 | 0.290 |
| CHD NSQIP Classification | 3.31 | 1.12–9.78 | 0.031 |
| Pre- and intraop PGE vs no | 1.51 | 0.54–4.24 | 0.437 |
| Preop loc: Floor vs outpatient | 0.54 | 0.06–4.93 | 0.581 |
| Preop loc: NICU vs outpatient | 1.07 | 0.26–4.50 | 0.924 |
| Preop loc: PCTU vs outpatient | 5.49 | 1.07–28.04 | 0.041 |
| Preop loc: PICU vs outpatient | 4.32 | 1.23–15.18 | 0.023 |
| Abbreviations: Congenital Heart Disease National Surgical Quality Improvement Program (CHD NSQIP), intraoperative (intraop), confidence interval (CI), Preoperative (Preop), location (loc), neonatial intensive care unit (NICU), Pediatric Cardiothoracic Unit (PCTU), Pediatric intensive care unit (PICU). | | | |
